# Photodynamic therapy accelerates skin wound healing through promoting re-epithelialization

**DOI:** 10.1093/burnst/tkab008

**Published:** 2021-09-06

**Authors:** Zengjun Yang, Xiaohong Hu, Lina Zhou, Yaxiong He, Xiaorong Zhang, Jiacai Yang, Zhenyu Ju, Yih-Cherng Liou, Han-Ming Shen, Gaoxing Luo, Michael R Hamblin, Weifeng He, Rui Yin

**Affiliations:** Department of Dermatology, Southwest Hospital, Third Military Medical University (Army Medical University), No. 30 Gaotanyan Street, Shapingba District, Chongqing, 400038, China; State Key Laboratory of Trauma, Burn and Combined Injury, Institute of Burn Research, Southwest Hospital, Third Military Medical University (Army Medical University), No. 30 Gaotanyan Street, Shapingba District, Chongqing, 400038, China; Chongqing Key Laboratory for Disease Proteomics, No. 30 Gaotanyan Street, Shapingba District, Chongqing, 400038, China; Department of Endocrinology, Southwest Hospital, Third Military Medical University (Army Medical University), No. 30 Gaotanyan Street, Shapingba District, Chongqing, 400038, China; Department of Dermatology, Southwest Hospital, Third Military Medical University (Army Medical University), No. 30 Gaotanyan Street, Shapingba District, Chongqing, 400038, China; State Key Laboratory of Trauma, Burn and Combined Injury, Institute of Burn Research, Southwest Hospital, Third Military Medical University (Army Medical University), No. 30 Gaotanyan Street, Shapingba District, Chongqing, 400038, China; Chongqing Key Laboratory for Disease Proteomics, No. 30 Gaotanyan Street, Shapingba District, Chongqing, 400038, China; State Key Laboratory of Trauma, Burn and Combined Injury, Institute of Burn Research, Southwest Hospital, Third Military Medical University (Army Medical University), No. 30 Gaotanyan Street, Shapingba District, Chongqing, 400038, China; Chongqing Key Laboratory for Disease Proteomics, No. 30 Gaotanyan Street, Shapingba District, Chongqing, 400038, China; Key Laboratory of Regenerative Medicine of Ministry of Education, Institute of Aging and Regenerative Medicine, Jinan University, No. 601 Huangpu Street, Tianhe District, Guangzhou, Guangdong Province, 510632, China; Department of Biological Sciences, National University of Singapore, 14 Science Drive 4, 117543, Singapore; Department of Physiology, Yong Loo Lin School of Medicine, National University of Singapore, 14 Science Drive 4, 117543, Singapore; State Key Laboratory of Trauma, Burn and Combined Injury, Institute of Burn Research, Southwest Hospital, Third Military Medical University (Army Medical University), No. 30 Gaotanyan Street, Shapingba District, Chongqing, 400038, China; Chongqing Key Laboratory for Disease Proteomics, No. 30 Gaotanyan Street, Shapingba District, Chongqing, 400038, China; Wellman Center for Photomedicine, Massachusetts General Hospital, Harvard Medical School, 40 Blossom Street, Boston, MA, 02114, USA; Laser Research Centre, Faculty of Health Science, University of Johannesburg, Doornfontein 2028, 17011 South Africa; State Key Laboratory of Trauma, Burn and Combined Injury, Institute of Burn Research, Southwest Hospital, Third Military Medical University (Army Medical University), No. 30 Gaotanyan Street, Shapingba District, Chongqing, 400038, China; Chongqing Key Laboratory for Disease Proteomics, No. 30 Gaotanyan Street, Shapingba District, Chongqing, 400038, China; Department of Dermatology, Southwest Hospital, Third Military Medical University (Army Medical University), No. 30 Gaotanyan Street, Shapingba District, Chongqing, 400038, China

**Keywords:** Photodynamic therapy, Wound healing, 5-aminolevulinic acid, Re-epithelialization, Epidermal stem cells, Transient amplifying cells

## Abstract

**Background:**

Epidermal stem cells (EpSCs) that reside in cutaneous hair follicles and the basal layer of the epidermis are indispensable for wound healing and skin homeostasis. Little is known about the effects of photochemical activation on EpSC differentiation, proliferation and migration during wound healing. The present study aimed to determine the effects of photodynamic therapy (PDT) on wound healing *in vivo* and *in vitro*.

**Methods:**

We created mouse full-thickness skin resection models and applied 5-aminolevulinic acid (ALA) for PDT to the wound beds. Wound healing was analysed by gross evaluation and haematoxylin–eosin staining *in vivo*. In cultured EpSCs, protein expression was measured using flow cytometry and immunohistochemistry. Cell migration was examined using a scratch model; apoptosis and differentiation were measured using flow cytometry.

**Results:**

PDT accelerated wound closure by enhancing EpSC differentiation, proliferation and migration, thereby promoting re-epithelialization and angiogenesis. PDT inhibited inflammatory infiltration and expression of proinflammatory cytokines, whereas the secretion of growth factors was greater than in other groups. The proportion of transient amplifying cells was significantly greater *in vivo* and *in vitro* in the PDT groups. EpSC migration was markedly enhanced after ALA-induced PDT.

**Conclusions:**

Topical ALA-induced PDT stimulates wound healing by enhancing re-epithelialization, promoting angiogenesis as well as modulating skin homeostasis. This work provides a preliminary theoretical foundation for the clinical administration of topical ALA-induced PDT in skin wound healing.

Highlights

- The rate and quality of wound healing is an active area of research.
- Photodynamic therapy might improve wound healing.
- Photodynamic therapy enhanced epidermal stem cell differentiation and migration.
- Epidermal stem cells are the basis for re-epithelialization and this is promoted by photodynamic therapy.

## Background

Wound healing is a well-organized physiological process requiring a complex interplay of resident mesenchymal and epithelial cells to perform the 4 stages of the process: haemostasis, inflammation, re-epithelialization and remodelling [1]. These stages overlap spatiotemporally and interact in complex ways to ultimately complete the healing process. Wound healing is an active area of clinical research, especially concerning factors that improve the healing rate or quality. It is critical to identify efficient, fast acting and economical treatments without side effects to enhance wound healing.

Epidermal stem cells (EpSCs), located in the hair follicle and interfollicular epidermis, participate in maintaining the homeostatic functions of the skin [2]. EpSCs and their daughter cells maintain the normal cutaneous structure by first migrating from their quiescent niches and then undergoing proliferation and differentiation [3]. In the case of skin damage, activated EpSCs are recruited to the epidermis and migrate towards the centre of the wound linearly, resulting in re-epithelialization of the wound and regeneration of the intact epidermis [4].

Photodynamic therapy (PDT) has been used to treat cancers, infectious diseases and inflammatory conditions, including acne vulgaris, rosacea, genital warts and others [5]. PDT uses a combination of a nontoxic dye, known as a photosensitizer, and specific wavelengths of harmless light, thereby photochemically generating reactive oxygen species (ROS) that affect cell signalling and selectively produce cell damage or death in target tissues and microorganisms [6]. The main products of photochemical activation are ROS, which mediate the regulation of intracellular signal transduction *in vivo*. Although the exact details of the regulatory process have not yet been elucidated, it is clear that ROS target signal transduction systems from the cell surface to the nucleus [7].

PDT dose is a critical factor in determining ROS concentration during photochemical reactions [8]. As opposed to the cellular toxicities caused by high levels of ROS, low-dose PDT influences proliferation and differentiation without significantly increased cell death [9], consequently promoting the differentiation of pluripotent stem cells, such as mesenchymal stem cells, osteoblast precursor cells [8], neural stem cells [10] and others. The regulatory effects of exogenous ROS on stem cells have been demonstrated *in vitro* [10]. Recently, a study demonstrated that *in situ* ROS production in murine skin activated hair follicle stem cell proliferation, stimulating hair growth in the quiescent phase and promoting burn healing [11]. Nevertheless, little is currently known about the precise effects of exogenous ROS produced by photodynamic activation on EpSCs during wound healing. We hypothesized that lower doses of PDT than those traditionally used to kill cancer cells might produce sufficient ROS to stimulate EpSCs. We tested and verified the idea *in vivo* and *in vitro* and found that low-dose PDT did enhance EpSC differentiation and migration that was helpful for the wound healing process, without significant cytotoxicity, by promoting re-epithelialization, angiogenesis and inflammatory regulation.

## Methods

### Isolation and culture of EpSCs

Mouse EpSCs were acquired from the skin of newborn (day 0–2) mice as previously described [12]. In brief, the separated mouse skin was incubated in 0.5% neutral protease (Roche, Switzerland) at 4°C overnight. The epidermis was carefully separated from the dermis and digested with trypsin (Gibco, USA). Then, the single isolated cells were suspended in keratinocyte serum-free medium (Invitrogen, USA) complemented with CaCl_2_ (0.05 mM), cholera toxin (10 M; Sigma, USA), streptomycin/penicillin (100 IU/ml) and mouse epidermal growth factor (10 ng/ml; Becton Dickinson Biosciences, USA) and cultured at 37°C in 5% CO_2_. The Animal Ethics Committee of the Third Military Medical University approved all protocols involving animals. Identification of the primary cells was achieved using flow cytometry and staining with the following fluorochrome-labelled murine monoclonal antibodies: α6-integrin (CD49f) (Invitrogen, USA), transferrin receptor (CD71) (Becton Dickinson Biosciences, USA) and keratin 14 (K14), keratin 15 (K15) and keratin 10 (K10) (all from Santa Cruz Biotechnology, USA).

### Preparation of the experimental animal model

We used C57BL/6(C57) mice (male, 18–20 g, specific pathogen-free) at 6 weeks of age. Mice were anaesthetized and shaved before induction of excisional wounds. After disinfecting with 75% alcohol, 2 excisional wounds were created by cutting down to the musculus panniculus carnosus with a sterile round skin biopsy punch (6 mm diameter) on the dorsal surface, with the 2 wounds at least 15 mm apart. The wound area was measured by tracing the wound margin and was calculated based on the photographs taken with a digital camera after the treatment using Image-Pro Plus (IPP) 6.0 software (Media Cybernetics, USA). The rate of wound healing was calculated as follows: healing rate (%) = (WA_i_−WA_n_)/WA_i_ × 100%, where WA_i_ represents the initial wound area and WA_n_ represents the residual wound area on the nth day post-surgery.

### PDT *in vivo* and *in vitro*

We used freshly prepared 5-aminolevulinic acid (ALA) (Shanghai Fudan-Zhangjiang Bio-Pharmaceutical Co. Ltd, China). For the i*n vivo* experiment, application of PDT was performed 24 hours after induction of the excisional wounds [13]. We applied a 3% ALA solution to the wounds with a 1 cm margin occluded with a polyurethane dressing (3 M Healthcare, USA). To implement PDT, a red light (635 nm) was applied to the dorsal wound surface after 3 hours of incubation in darkness for a total dose of 6 J/cm^2^ using a light-emitting diode (LED) lamp (Omnilux Revive, Photo Therapeutics, Inc., UK). Animals in the irradiation group received only red light and those in the ALA group received only ALA; control group animals underwent the natural recovery process. None of the animals were exposed to light for at least 48 hours after the treatment.

For the *in vitro* experiment, cultured EpSCs were randomly divided into 4 groups, namely the control, irradiation, ALA and PDT groups. EpSCs were incubated with 0.1 mM ALA for 6 hours in a serum-free cell culture medium. In the PDT group, the cells were washed 3 times with phosphate-buffered saline and immediately irradiated with an LED light (635 nm) at a dose of 4 J/cm^2^ in a serum-free medium. After irradiation, EpSCs were cultured in fresh medium for further assays.

### EpSC apoptosis assay *in vitro*

A total of 5 × 10^5^ EpSCs were collected by centrifuging for 10 minutes at 4°C after dissociation with 0.25% trypsin. Following the manufacturer’s protocol, cells were stained with Annexin V-FITC and propidium iodide (Invitrogen, USA) and detected using flow cytometry. The percentage of apoptotic cells was measured using FlowJo software (Tree Star Inc., USA).

### Scratch wound migration assay *in vitro*

An *in vitro* wound healing model was used to study EpSC motility in 6-well plates, as previously reported [14]. Briefly, EpSCs were cultured to near confluence in 6-well plates and incubated in a complete medium supplemented with 0.1 mM ALA and 4 μg/mL mitomycin C (Sigma, USA) for 6 hours. The scratch wounds were created in the monolayer (0 hours) with a sterile 200 μL pipette tip. The cells were illuminated by red light and cultured with a fresh culture medium post-PDT. They were monitored for 48 hours using an inverted phase microscope. The control groups included light control, dark control and normal control. The scratch areas were quantified using IPP 6.0 software. The rate of migration was calculated as follows: rate of migration (%) = (initial area−residual area)/initial area × 100%.

### ROS detection

ROS production in the wound during PDT was evaluated *in vivo* using ROS-sensitive 2′,7′-dichlorodihydrofluorescein diacetate (DCFH-DA) (Sigma, USA) [11]. After preparation of excisional wounds and PDT application as previously described (incubation of treated animals for 3 hours in the dark, followed by irradiation with 6 J/cm^2^ of 635 nm light), we topically applied 50% ethanol (1 mg/ml) with DCFH-DA on wound areas 20 minutes after irradiation. ROS levels generated in the wound area were evaluated using an In Vivo Imaging System (IVIS) Spectrum (Xenogen, CA) 45 minutes after irradiation. The filter settings were 465 nm for excitation and 560 nm for emission. The ROS-related green emission was also evaluated under a fluorescence microscope using frozen sections.

### Histological examination

On day 5 post-surgery, mice were sacrificed and the harvested wound tissues were carefully removed. Haematoxylin–eosin (HE) staining and histological analyses were performed as previously reported [14]. The length of the neo-epidermis and the granulation thickness were measured using IPP 6.0 software. Immunohistochemistry (IHC) staining was undertaken using the following antibodies: anti-insulin-like growth factor-1 (IGF-1) (1:200), anti-interleukin (IL)-1 (1:50), anti-IL-23 (1:400), anti-tumour necrosis factor-α (TNF-α) (1:300), anti-platelet endothelial cell adhesion molecule (CD31) (1:100), anti-vascular endothelial growth factor A (VEGFA) (1:100) and anti-proliferating cell nuclear antigen (PCNA) (1:200) (all from Abcam, UK).

### Statistical analysis

All data are displayed as mean ± standard deviation. Statistical differences were calculated using one-way analysis of variance for comparisons among multiple groups simultaneously, followed by the least significant difference test. Two-way analysis of variance was used to compare multiple time points using SPSS 20 software (IBM, USA). The significance level was set at *p* < 0.05.

## Results

### PDT induces ROS production around skin wound regions

**Figure 1.**
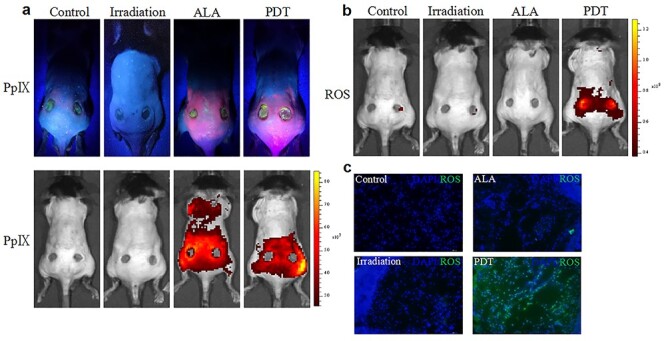
5-aminolevulinic acid (ALA) photodynamic therapy (PDT) switches on *in situ* reactive oxygen species (ROS) production in skin wound regions. **(a)** Accumulation of protoporphyrin IX (PpIX) induced by topical treatment with ALA in skin wound regions as compared to control groups. **(b, c)** PpIX-dependent ROS production monitored by 2′,7′-dichlorodihydrofluorescein diacetate. *DAPI* 2-(4-Amidinophenyl)-6-indolecarbamidine dihydrochloride

We measured the accumulation of protoporphyrin IX (PpIX) in the wound regions after ALA solution application. We found that topical application of ALA on the skin wounds resulted in significant absorption of PpIX around the wounds, as demonstrated by reddish-pink fluorescence. The fluorescent signal emitted by PpIX was markedly enhanced when measured using the IVIS living imaging system (Figure 1a). Subsequent irradiation of treated skin with red light (635 nm) promoted transient production of ROS in the tissue of wounds and margin areas (Figure 1b, c).

### ALA-induced PDT promoted skin wound closure and wound re-epithelialization

**Figure 2.**
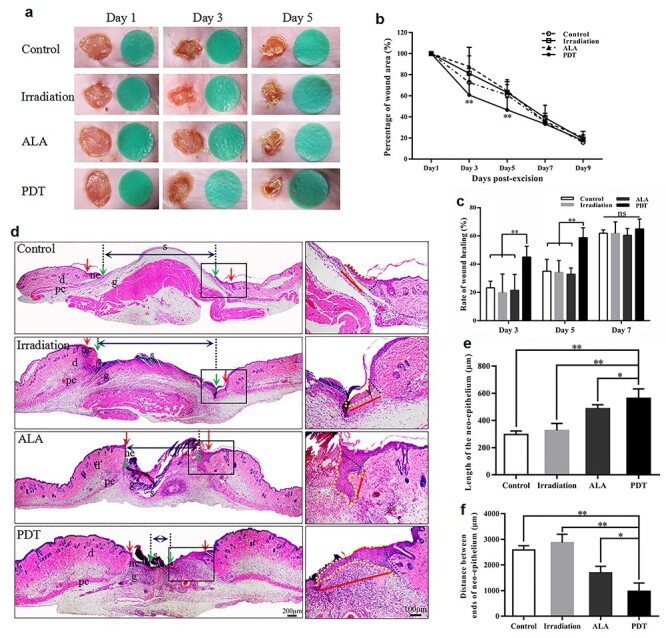
5-aminolevulinic acid (ALA) photodynamic therapy (PDT) promotes wound healing and wound re-epithelialization. **(a)** The macroscopic appearance of the wounds at different time points. **(b, c)** Wound healing at different time points (n = 5 per group). **(d)** The newly formed epithelium in the wound tissue at day 5 post-wounding in different groups after haematoxylin–eosin staining (blue double-ended arrows represent the distance between the epithelial tips; red arrows indicate wound edges; green arrows indicate tips of epithelial tongues; red lines indicate the neo-epidermis). Scale bars: 200 μm (left) and 100 μm (right). **(e)** Quantification of the neo-epidermal length (n = 5 per group). **(f)** Quantification of the distance between the epithelial tips (n = 5 per group). Statistical analysis: ^**^p < 0.01, ^*^p < 0.05. *pc* panniculus carnosus, *d* dermis, *ne* neo-epithelium, *g* granulation tissue, *s* scab

We found that the wounds appeared to be clean and new epidermis could be observed at the edge of the wounds on days 3 and 5 in the PDT group compared with the other 3 groups (Figure 2a). The wound healing rate in the experimental groups was significantly higher than in the other 3 groups on days 3 and 5 (Figure 2b, c). These macroscopic findings were highly consistent with histological observations. On day 5 post-surgery the wounds of the control mice were covered with fibrin-rich clots, whereas the clot had been replaced by cellular and vascularized granulation tissue in PDT animals (Figure 2d).

Wound re-epithelialization was evaluated by measuring the length of the new epidermis in the wounds using HE staining. The average length of the neo-epithelium in the PDT group was notably longer than that of the other groups. The average lengths of the neo-epithelium on day 5 in the control, irradiation, ALA and PDT groups were 296.63 μm, 326.91 μm, 486.04 μm and 563.34 μm, respectively (PDT *vs* control, *p* < 0.01; PDT *vs* irradiation, *p* < 0.01; and PDT *vs* ALA, *p* < 0.05) (Figure 2d, e). Likewise, the longitudinal diameters between the tips of the epithelial tongues were dramatically shorter in the PDT group than in the other groups on day 5 after wounding (PDT, 972.87 μm *vs* control, 2584.62 μm, *p* < 0.01; PDT, 972.87 μm *vs* irradiation, 2871.71 μm, *p* < 0.01; and PDT, 972.87 μm *vs* ALA, 1692.68 μm, *p* < 0.05) (Figure 2f).

### The effect of ALA-induced PDT on EpSC differentiation, migration and apoptosis

**Figure 3.**
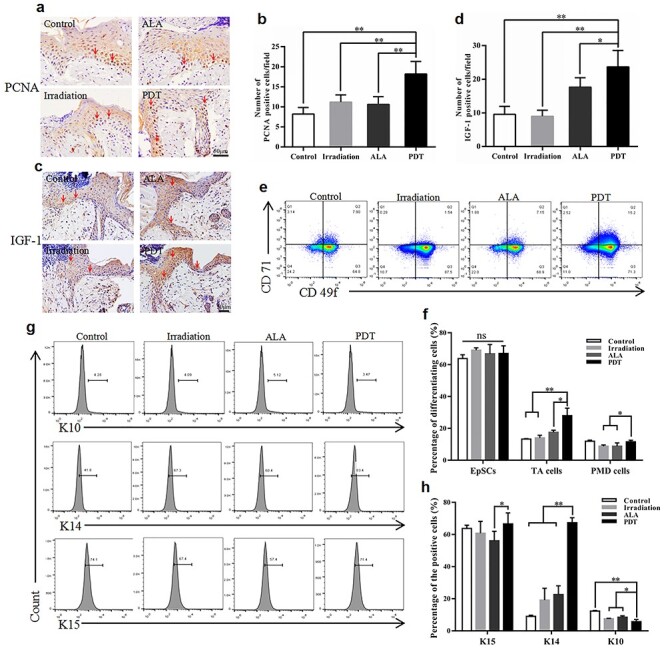
5-aminolevulinic acid (ALA) photodynamic therapy (PDT) promotes the proliferation and differentiation of epidermal cells. **(a, c)** Typical images of immunohistochemistry staining of proliferating cell nuclear antigen (PCNA) and insulin-like growth factor-1 (IGF-1) at day 5 post-surgery. The red arrows indicate PCNA positive and IGF-1 positive keratinocytes. Scale bars: 50 μm. Quantitative counts of **(b)** PCNA positive keratinocytes and **(d)** IGF-1 positive keratinocytes per field (500 × 500 pixels/field). The percentages of epidermal stem cells (EpSCs), transient amplifying cells (TA cells) and post-mitotic differentiating cells (PMD cells) in the epidermis at wound margins on day 3 post-wounding were detected with **(e)** staining for surface biological markers and **(g)** intracellular markers using flow cytometry. **(f, h)** Quantitation of the epidermal cells characterized by both sets of biological markers. Statistical analysis: ^**^p < 0.01, ^*^p < 0.05. *ns* no significance, *CD71* transferrin receptor, *CD49f* α6-integrin, *K10* keratin 10, *K14* keratin 14, *K15* keratin 15

Re-epithelialization is closely regulated by the proliferation and differentiation of epidermal cells. We reasoned that the positive effect of low-dose ALA-induced PDT on cutaneous cells, particularly epidermal cells, would produce a physiological response. To investigate the proliferation of keratinocytes *in vivo*, we measured expression levels of PCNA, a biomarker for cell proliferation, in the neo-epidermis around the wound using IHC staining. We found that PDT appeared to cause a significantly greater number of PCNA positive cells at the wound margin than the other groups at day 5 (Figure 3a, b). Due to the critical role of IGF-1 in the proliferation of epidermal cells, we measured the production of IGF-1 in the neo-epidermis. IGF-1 production was markedly higher in the epidermis around the wounds of the PDT group than in the other 3 groups based on the IHC analysis on day 5 (Figure 3c, d).

**Figure 4.**
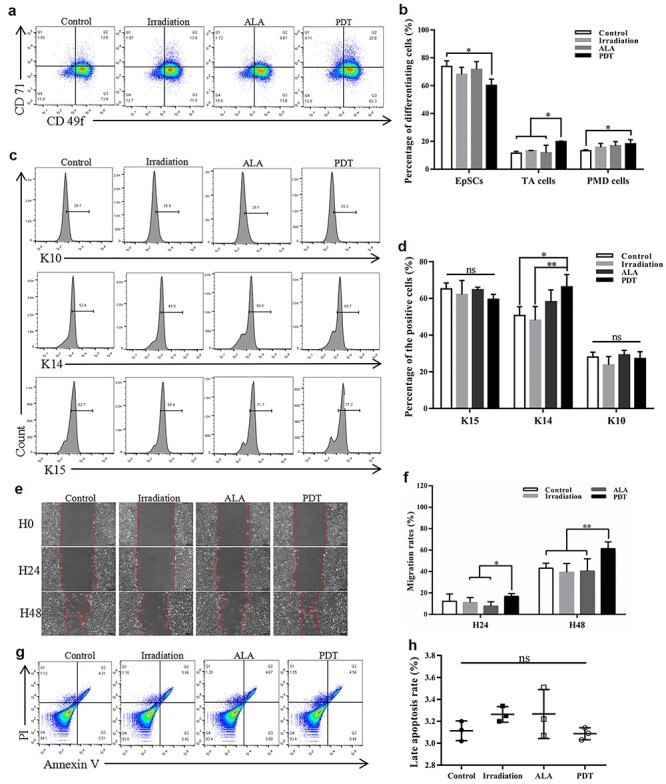
The effect of 5-aminolevulinic acid (ALA) photodynamic therapy (PDT) on epidermal stem cell (EpSC) differentiation, migration and apoptosis *in vitro*. Cultured EpSCs were treated with different conditions according to the groups. The levels of EpSC surface markers **(a, b)** and intracellular markers **(c, d)** were analysed using flow cytometry 24 hours post-treatment. **(e)** Typical images of cell migration and recovery in scratched areas at 24 hours (H24) and 48 hours (H48). The red dashed line shows the leading edge of migrating cells. Scale bars: 100 μm. **(f)** Quantitation of the remaining area that has not yet been covered (n = 6 per group). **(g)** Apoptosis was evaluated using Annexin V/3,8-Diamino-5-[3-(diethylmethylammonio) propyl]-6-phenylphenanthridinium diiodide (PI) staining. **(h)** Quantitation of the late apoptosis rate. Statistical analysis: ^**^p < 0.01, ^*^p < 0.05. *CD71* transferrin receptor, *CD49f* α6-integrin, *TA cells* transient amplifying cells, *PMD cells* post-mitotic differentiating cells, *K10* Keratin 10, *K14* Keratin 14, *K15* Keratin 15, *H0* 0 hour, *ns* no significance

**Figure 5.**
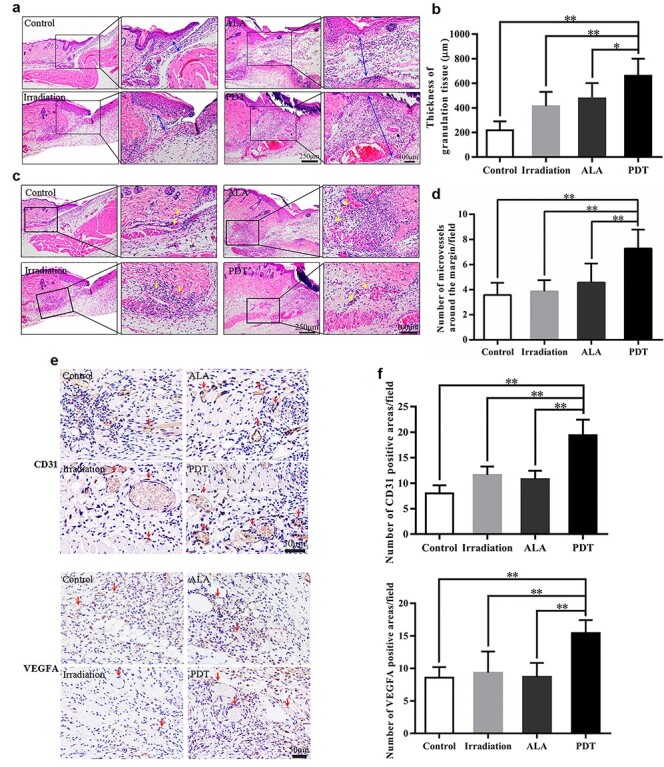
5-aminolevulinic acid (ALA) photodynamic therapy (PDT) promotes angiogenesis at the wound edge. **(a, c)** Representative haematoxylin–eosin stained images of the wound edge for each group on day 5. The blue double-ended arrows indicate granulation tissue and the yellow arrows indicate micro-vessels around the margin. Scale bars: 250 μm (left) and 100 μm (right). Quantitative determination of **(b)** thickness of granulation tissue and **(d)** number of micro-vessels around the wound margin in different groups. **(e, f)** Immunohistochemistry (IHC) analysis of wound sections on day 5 post-operation, **(e)** Typical images of IHC staining for platelet endothelial cell adhesion molecule-1 (CD31) and vascular endothelial growth factor A (VEGFA). The red arrows indicate the positive area. Scale bars: 50 μm. **(f)** Quantitative counts of CD31 positive and VEGFA positive areas per field (800 × 800 pixels/field). Statistical analysis: ^**^p < 0.01, ^*^p < 0.05

**Figure 6.**
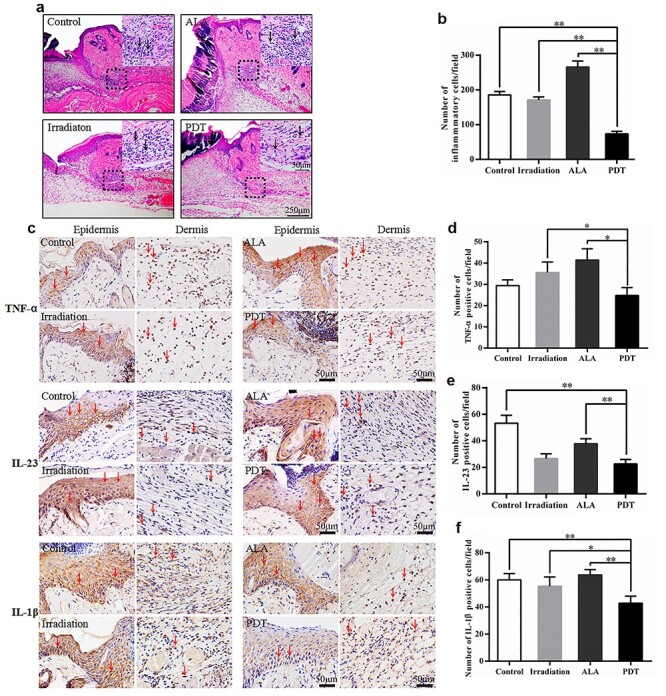
5**-**aminolevulinic acid (ALA) photodynamic therapy (PDT) inhibits the inflammatory response. **(a)** Representative haematoxylin–eosin stained images of the injury site for each group. The black arrows indicate inflammatory cells. Scale bars: 250 μm (main images) and 50 μm (insets). **(b)** Quantitative determination of the number of inflammatory cells for each group (500 × 500 pixels/field). **(c–f)** Immunohistochemistry (IHC) analysis of skin wound sections at day 5 post-operation. **(c)** Representative images of IHC staining of tumour necrosis factor-α (TNF-α), interleukin-23 (IL-23) and interleukin-1β (IL-1β). The red arrows indicate the positive cells. Scale bars: 50 μm. Quantitative counts of **(d)** TNF-α positive cells, **(e)** IL-23 positive cells and **(f)** IL-1β positive cells per field (500 × 500 pixels/field). Statistical analysis: ^**^p < 0.01, ^*^p < 0.05

The impact of ALA-induced PDT on the differentiation of epidermal cells at the wound edge was further evaluated using flow cytometry. The proportion of transient amplifying cells (TA cells) characterized by the CD49f-bright/CD71-bright (CD49f^bri^CD71^bri^) phenotype was significantly higher in the PDT group than the other groups (PDT, 27.98% *vs* control, 13.32%, *p* < 0.01; PDT, 27.98% *vs* irradiation, 14.05%, *p* < 0.01; and PDT, 27.98% *vs* ALA, 17.58%, *p* < 0.05) (Figure 3e, f). The same trend was observed in post-mitotic differentiating cells (PMD cells) with the CD49f-dim (CD49f^dim^) phenotype, with significantly higher proportions in the PDT group than in the light control and dark control groups. However, no obvious differences were found between each group for EpSCs with the CD49f^bri^CD71^dim^ phenotype. The expression of K10, K14 and K15, another set of biological markers used to define the differentiation state of keratinocytes, was measured using flow cytometry with intracellular cytokine staining. Figure 3g, h shows that the expression of K14 was dramatically higher in the PDT group than in the control groups (*p* < 0.01). The opposite result was observed for the expression of K10, with a significant decrease in the PDT group. Expression levels of K15 in the PDT mice were significantly higher than in the ALA group. However, no significant differences were observed compared to the blank control or light groups (Figure 3g, h).

Differentiation, proliferation and migration of EpSCs are the cellular bases for cutaneous re-epithelialization. We investigated the potential influence of low-dose ALA-induced PDT on EpSCs *in vitro*. PDT significantly increased the proportion of TA cells compared with the other groups, which was in line with the *in vivo* result. Precisely the opposite occurred in EpSCs, with a significant decrease in the PDT group (Figure 4a, b). In contrast, compared with the control and light groups, the expression of K14 in EpSCs was significantly greater in the PDT group (Figure 4c, d). The impact of PDT on the migratory capacity of EpSCs was measured using the scratch assay (Figure 4e, f). The migration of the isolated EpSCs was not significantly higher in the ALA-induced PDT group than in the other groups (Figure 4e, f). There was no apparent effect of PDT on apoptosis among the 4 groups (Figure 4g, h).

### Granulation tissue formation and angiogenesis in the dermis

Granulation tissue formation and angiogenesis are the bases of efficient wound repair. The effect of ALA-induced PDT was evaluated using HE staining and IHC analysis. ALA-induced PDT increased granulation tissue remodelling, as the average thickness in PDT mice was 661.03 μm on the fifth day, which was much thicker than that in the control, irradiation and ALA groups (217.57 μm, 413.12 μm and 476.62 μm, respectively; PDT *vs* control, *p* < 0.01; PDT *vs* irradiation, *p* < 0.01; and PDT *vs* ALA, *p* < 0.05) (Figure 5a, b). Furthermore, the average number of neo-capillaries at the wound edge was significantly greater post-ALA-induced PDT treatment when compared with other groups (PDT, 7.29 *vs* control, 3.57, *p* < 0.01; PDT, 7.29 *vs* irradiation, 3.85, *p* < 0.01; and PDT, 7.29 *vs* ALA, 4.57, *p* < 0.01) (Figure 5c, d).

To confirm these results, expression levels of CD31, a marker of angiogenesis, were measured using IHC. The growth of neo-vessels into the wounds was significantly greater in the PDT group, consistent with the observations on HE staining (Figure 5e, f). Because VEGFA is a critical growth factor for angiogenesis, we further examined its production. Compared with the control, irradiation and ALA groups, we observed that expression levels of VEGFA were dramatically higher in the wound tissue of the PDT group at day 5 after PDT (Figure 5e, f).

### The expression of proinflammatory cytokines and inflammatory infiltrate during wound healing

Inflammatory responses establish the first initial host defense against wounds and play critical roles in the entire healing process [15]. Moderate inflammation is advantageous to the normal healing process, while excessive inflammation is harmful. To evaluate the immunomodulatory effect of ALA-induced PDT on wound repair, we analysed the inflammatory cell infiltration and cytokine expression in mouse skin wound tissue. ALA-induced PDT dramatically reduced inflammatory cell infiltration at day 5, as the average value was less in PDT mice than in the other groups (PDT, 73.40 *vs* control, 185.40, *p* < 0.01; PDT, 73.40 *vs* irradiation, 170.80, *p* < 0.01; and PDT, 73.40 *vs* ALA, 265.60, *p* < 0.01) (Figure 6a, b). There were significantly lower levels of proinflammatory cytokines, including IL-23, IL-1β and TNF-α, in the wound tissue of PDT mice than in the control, irradiation and ALA groups (Figure 6c, d e, f).

## Discussion

Low-dose ALA-induced PDT accelerated wound closure in an acute mouse skin excision model, as demonstrated by the induction of re-epithelialization, neo-vascularization, granulation and inflammatory infiltration. In addition, the length of the neo-epithelium and the distance between the epithelial tongue tips were improved by ALA-induced PDT.

Healing acute injuries demands a rapid and efficient re-epithelialization process to recover cutaneous integrity [16,17]. Cutaneous healing requires expansion of the neo-epithelium across the wound bed [18], a process sustained by EpSCs that arise from niches found in the interfollicular epidermis and hair follicles [19,20]. In response to injury, EpSCs play a pivotal role in the re-epithelialization process. They are transiently activated to proliferate and differentiate into migratory cells from their otherwise habitually quiescent niches [21]. The re-epithelialization process during the proliferation phase of wound repair requires the activation of quiescent EpSCs [22], their proliferation and migration over the site of injury and their differentiation into a stratified epidermis. Activated EpSCs show upregulation of PCNA (Figure 3a) and are divided into 3 subtypes—CD49f^bri^CD71^dim^, CD49f^bri^CD71^bri^ and CD49f^dim^—with the characteristics of EpSCs, TA cells and PMD cells, respectively [23–25]. Under homeostatic conditions, the repair and maintenance of stratified skin epithelium relies on epithelial progenitor cells, including both EpSCs and TA cells [23,26]. TA cells are the progeny cells of EpSCs by asymmetric division with a proliferative capacity and have been identified as a pool of rapidly proliferating cells [3,27]. We found that ALA-induced PDT appeared to increase the proportion of TA cells characterized by both sets of biological markers (Figure 3e, f, g, h).

Successful stratification and differentiation of the neo-epithelium are essential for regaining the protective skin barrier after wound closure [28]. As previously reported, K14-positive cells migrate into the wound bed, followed by a mixed population of supra-basal keratinocytes expressing various inducible keratins during acute wound healing [26,29]. However, no experiments have yet been performed to define the role of ALA-induced PDT on EpSCs *in vivo*. The protein expression levels of K10 were lower in PDT mice, suggesting that K10-positive cells remain at the wound margin and contribute essentially nothing to the formation of the neo-epithelium [29]. These results indicate that ALA-induced PDT might enhance re-epithelialization by improving EpSC proliferation, differentiation and migration. Li *et al.* reported that activated dendritic epidermal T cells around the wound edge secreted IGF-1 to contribute to the formation of neo-epithelium, thereby accelerating the healing process [30]. In the present study, IGF-1 expression was greater in the PDT group, suggesting that ALA-induced PDT stimulates IGF-1 expression to accelerate wound healing (Figure 3c, d). The *in vivo* results inspired us to perform *in vitro* experiments to investigate the effect of ALA-induced PDT on cultured EpSCs. We found that ALA-induced PDT enhanced EpSC migration (Figure 4e, f), the proportion of TA cells (Figure 4a, b) and the expression of K14 (Figure 4c, d), suggesting enhanced cell differentiation post-ALA-induced PDT treatment.

Angiogenesis is also an essential factor for accelerated wound healing. Garcia *et al.* described the positive effects of PDT on proliferation and angiogenesis in burns [31]. We found that wounds in PDT mice displayed thicker granulation tissue and more neo-capillaries (Figure 5a, b, c, d). Thus, enhanced angiogenesis and granulation tissue formation could also help explain the rapid closure of the wounds [32]. On day 5 post-treatment, low-dose PDT promotes the expression of VEGFA, a key growth factor for angiogenesis in wound tissue (Figure 5e, f) [33,34]. It also reduced the inflammatory infiltrates and there were relatively lower expression levels of proinflammatory cytokines, including IL-23, IL-1β and TNF-α (Figure 6). Thus, we speculate that ALA-induced PDT inhibits proinflammatory cytokines and inflammatory infiltrates at the middle stage of wound healing.

## Conclusions

Low-dose ALA-induced PDT activates cell proliferation, angiogenesis and regulates skin homeostasis, promoting wound healing by activating *in situ* ROS production. Our study is the first to focus on the role of EpSCs in ALA-induced PDT-stimulated wound healing. Our findings suggest that topical low-dose ALA-induced PDT might be used clinically for skin wound healing.

## Abbreviations

ALA: 5-aminolevulinic acid; CD31: platelet endothelial cell adhesion molecule-1; CD49f: α6-integrin; CD71: transferrin receptor; DCFH-DA: 2′,7′-dichlorodihydrofluorescein diacetate; EpSCs: epidermal stem cells; HE: haematoxylin–eosin; IGF-1: insulin-like growth factor-1; IHC: immunohistochemistry; IL: interleukin; IPP: Image-Pro Plus; IVIS: In Vivo Imaging System; K10: keratin 10; K14: keratin 14; K15: keratin 15; PCNA: proliferating cell nuclear antigen; PDT: photodynamic therapy; PMD cells: post-mitotic differentiating cells; PpIX: protoporphyrin IX; ROS: reactive oxygen species; TA cells: transient amplifying cells; TNF-α: tumour necrosis factor-α; VEGFA: vascular endothelial growth factor A.

## Availability of data and materials

All data generated and/or analysed during the current study are included in this published article.

## Authors’ contributions

ZY: conceptualization, formal analysis, investigation, original draft preparation, writing of the original draft. XH: conceptualization, formal analysis, methodology. LZ: investigation, methodology. YH: investigation, visualization. XZ: investigation, resources. JY: resources. ZJ: conceptualization, methodology. YL: conceptualization. HS: visualization, supervision. GL: project administration, methodology. MRH: writing, review and editing. WH: funding acquisition, writing of the original draft. RY: funding acquisition, study design, writing, review and editing. All authors read and approved the final manuscript.

## Funding

This work was supported by National Natural Science Foundation of China (grant No. 81571902 and 31872742).

## Ethics approval and consent to participate

All protocols involving animals were approved by the Animal Ethics Committee of Third Military Medical University and performed in accordance with international animal welfare standards.

## Conflicts of interest

The authors declare that they have no competing interests.
